# Personalised medicine through AI-enhanced integration of diagnostic imaging and radiation therapy

**DOI:** 10.1186/s41747-025-00664-0

**Published:** 2025-12-22

**Authors:** Silvia Bottazzi, Giuditta Chiloiro, Luca Russo, Anna Rame, Alessandra Iacono, Benedetta Gui, Luca Boldrini, Maria Antonietta Gambacorta, Evis Sala

**Affiliations:** 1 Dipartimento di Diagnostica per Immagini e Radioterapia Oncologica, Fondazione Policlinico Universitario Agostino Gemelli IRCCS, Rome, Italy; 2 Dipartimento di Scienze radiologiche ed ematologiche, Università Cattolica del Sacro Cuore, Rome, Italy

**Keywords:** Artificial intelligence, Magnetic resonance imaging, Precision medicine, Radiotherapy, Biomarkers (tumours)

## Abstract

**Abstract:**

The integration of diagnostic imaging with radiation therapy (RT) is evolving into a continuous workflow, significantly advancing personalised oncology care. Recent technological innovations, particularly the incorporation of real-time magnetic resonance imaging (MRI) with linear accelerators, have markedly enhanced RT precision, improving target coverage and reducing radiation exposure to surrounding healthy tissues. Furthermore, real-time MRI enables the collection of quantitative imaging data during each treatment fraction, potentially leading to the identification of quantitative imaging biomarkers. These biomarkers can capture dynamic biological changes during RT, offering unprecedented insights into treatment response. The integration of these imaging biomarkers with clinical, genomic, and pathological data into artificial intelligence (AI)-supported clinical decision support systems promises to further refine therapeutic personalisation. In this context, AI plays a central role by automating labour-intensive tasks, extracting quantitative metrics, and integrating multidimensional data into clinically meaningful predictive models. This review outlines a vision for the future of RT, highlighting how the synergy of advanced imaging, AI, and multidomain data through three logical steps: (1) rethinking and reorganising the patient care journey; (2) from imaging “for” to imaging “with” RT; and (3) incorporation into clinical decision support systems. This integration will support the development of personalised, biologically driven treatment strategies.

**Relevance statement:**

The longitudinal integration of diagnostic imaging and RT, facilitated by AI, could significantly enhance clinical workflow efficiency and therapeutic accuracy in oncology.

**Key Points:**

- Oncological care is transitioning from disease-centred to patient-centred, with tumour boards representing the junction for shared multidisciplinary decisions.
- Integrating advanced imaging with RT enables quantitative imaging biomarkers extraction that captures tumour changes throughout the course of treatment.
- Artificial intelligence plays a central role in automating resource-intensive processes and integrating large-scale multidomain data towards personalised medicine.

**Graphical Abstract:**

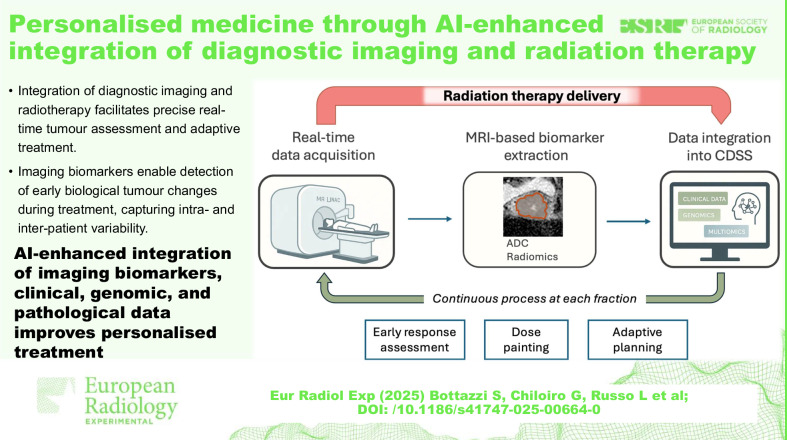

## Introduction

The integration of diagnostic imaging with radiation therapy (RT) is progressively evolving from isolated procedural steps to a cohesive, seamless workflow. Historically, imaging modalities such as computed tomography (CT), magnetic resonance imaging (MRI) and positron emission tomography (PET) have been employed primarily at discrete points—initial diagnosis, treatment planning, and post-treatment evaluation— with overall limited continuity across the patient journey [1, 2].

Recent advancements support the necessity for continuous, longitudinal integration of imaging throughout the RT pathway, aligning with a broader shift from a disease-centred to a patient-centred therapeutic model. Target definition strategies should move beyond morphological tumour characterisation to consider patient-specific biological variability and dynamic tumour response pathways to optimise therapy. This approach enables treatment personalisation by accounting for real-time variations in tumour biology and disease response. Its effective implementation requires close collaboration among radiation oncologists, radiologists, medical physicists, and RT technologists.

Such a personalised model necessitates the systematic acquisition and interpretation of large volumes of data from diverse sources, including imaging, genomics, pathology, and clinical records. The complexity and scale of this information exceed the capacity of traditional workflows. Therefore, artificial intelligence (AI) plays a central role, not only in automating labour-intensive processes, but also in extracting quantitative metrics and integrating multidimensional data into clinically meaningful predictive models.

**Fig. 1:**
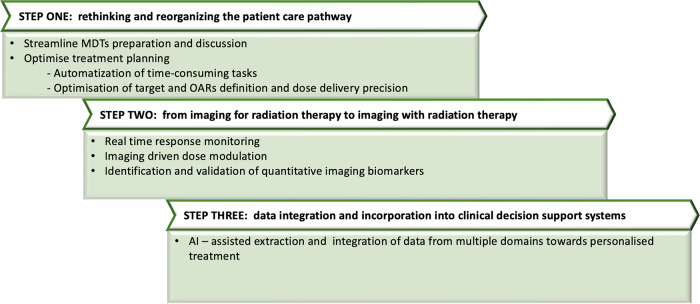
A structured, three-step workflow is presented: (1) reorganising patient care pathways via streamlined multidisciplinary team (MDT) processes and automation of treatment planning tasks; (2) evolving the imaging paradigm from initial planning to dynamic integration within radiation therapy, enabling real-time response assessment and targeted dose modulation through quantitative imaging biomarkers; (3) employing artificial intelligence (AI) to integrate multidomain data into clinical decision support systems. OARs, organs at risk

In the following sections, we outline a vision on the future of RT, focusing on how the integration of advanced imaging, AI, and multidomain data will support the development of biologically driven, individualised treatment strategies. This article is structured into three main sections, each representing a step towards the implementation of personalised medicine (Fig. 1).

## Step one: rethinking and reorganising the patient care journey

### Multidisciplinary tumour boards

Multidisciplinary tumour boards (MDTs) represent the first critical point of interdisciplinary contact in oncology, where case review, integration of expertise from different specialists, and shared discussion define the optimal patient management strategy [3]. Joint image review within MDTs enables accurate staging, assessment of operability, and determination of RT suitability [3, 4]. However, access to MDTs is largely limited to tertiary centres, where patients are typically referred only after multiple isolated consultations with different specialists. This process contributes to treatment initiation delays, currently averaging approximately 60 days [5], and simultaneously results in an exponential increase in the number of cases discussed at MDTs, demanding greater resources for preparation and discussion. AI-based MDT platforms and clinical decision support systems (CDSS) could help mitigate these challenges by reducing workload in tertiary centres and supporting other facilities in selecting optimal treatment strategies while ensuring adherence to best practices [6–8].

### Optimising treatment planning

Radiotherapy conventionally starts 1 or 2 weeks after the treatment decision, due to the inherently complex, multistep workflow. This interval introduces a risk of anatomical and physiological changes between diagnosis and treatment start, potentially leading to target volume variations [9]. A dedicated simulation CT scan is almost universally required to obtain electron density information, which is essential for accurate dose calculation, and treatment planning.

However, as the waiting time increases, the probability that the patient’s clinical condition—such as tumour progression, weight fluctuation, or anatomical alterations—will diverge from what was captured during the simulation CT, also increases. Consequently, this mismatch can result in discrepancies between the planned and the delivered dose distribution. Moreover, target volumes and organs at risk delineation remain a significant source of variability and one of the most time-consuming and error-prone steps in the RT workflow [10].

The advancement of deep learning algorithms has yielded promising results in automating tumour detection and segmentation during RT, reducing contouring time, minimising interobserver variability and decreasing clinician workload. These advantages are particularly relevant within the context of online adaptive RT, where accuracy and time-efficiency are critical [10–12]. These tools can support more precise and consistent longitudinal assessment of tumour volume and morphology throughout the course of treatment [13]. An emerging innovation is the All-in-One workflow, an AI-driven, fully automated process based on CT-integrated linear accelerators [14]. This model integrates simulation, autosegmentation, autoplanning, image guidance, beam delivery, and *in vivo* quality assurance into a single procedure conducted with the patient on the treatment couch. Compared with conventional workflows, All-in-One can significantly reduce time-to-treatment, improve workflow efficiency, and minimise anatomical variation [15]. Preliminary clinical experience, including nasopharyngeal cancer, supports its feasibility and potential for online adaptive replanning [16].

CT is the standard imaging modality for RT planning. However, the advent of MRI introduced significant advantages. MRI is the standard of care for the diagnosis, treatment response evaluation and recurrence detection for several cancer types (*e.g*., cervical, rectal, prostate and neuro-oncology) [17–20]. This is due to the superior soft tissue contrast—which allows for more precise identification and delineation of target volumes and organs at risk—and functional sequences allowing for a more precise evaluation of tumour response and differentiation from post-treatment changes such as inflammation and radionecrosis [17, 21–25]. Consequently, RT planning currently relies on a combined MRI-CT workflow. Target volumes and organs at risk are typically delineated on MRI due to its superior anatomical detail, whereas CT provides the electron density data required for dose calculation [26, 27].

However, this dual-modality approach necessitates accurate image registration between MRI and CT datasets. Despite its widespread use, the image registration between MRI and CT is frequently associated with geometric uncertainties that can significantly affect dose delivery precision, thereby impacting treatment efficacy. Since both the planning and delivery phases in an MRI-CT workflow are ultimately anchored to the anatomical framework defined by CT images, any misalignment or anatomical variation between MRI and CT acquisitions may require manual adjustments to the transferred contours [9].

Moreover, the need for close temporal proximity between MRI and CT acquisitions increases logistical complexity [28–30]. An MRI-only workflow mitigates these issues by eliminating the need for CT, avoiding registration-related uncertainties, simplifying the workflow, reducing overall workload and potentially lowering costs [26]. Additionally, MRI-only workflows could substantially decrease the duration of simulation procedures, with a potential treatment slot of approximately 15 min compared to the 30–50 min of conventional RT workflows [31]. Several non-AI strategies have attempted to bypass CT simulation, including the use of pregenerated atlases and bulk electron density assignment to predefined tissue classes (*e.g*., fat, water, lung, bone) [32–34]. However, comparative studies, particularly in pelvic and abdominal settings, have shown these methods to be generally inferior to deep learning-based approaches in both dose accuracy and image generation time [35, 36].

Synthetic CT (sCT) technology has emerged as a promising deep learning-based solution to enable MRI-only workflows by assigning HU values to MRI voxels, which are then converted to electron density values using calibration curves within the treatment planning system [28, 32, 37–39]. This facilitates accurate dose calculations equivalent to those derived from conventional CT, removing the need for separate CT imaging sessions [34, 40]. Their clinical implementation showed promising results, such as in cervical cancer, where deep learning-generated sCT enabled PET/MRI-based attenuation correction and dose planning within a unified workflow, and in prostate cancer, where sCT combined with semi-automatically burned-in fiducial markers achieved comparable accuracy to conventional CT/MRI workflows [41–43]. Despite its promise, sCT implementation remains variable across institutions, and current methods require integration into robust, standardised quality assurance protocols to ensure reliability and clinical safety [44, 45].

**Fig. 2:**
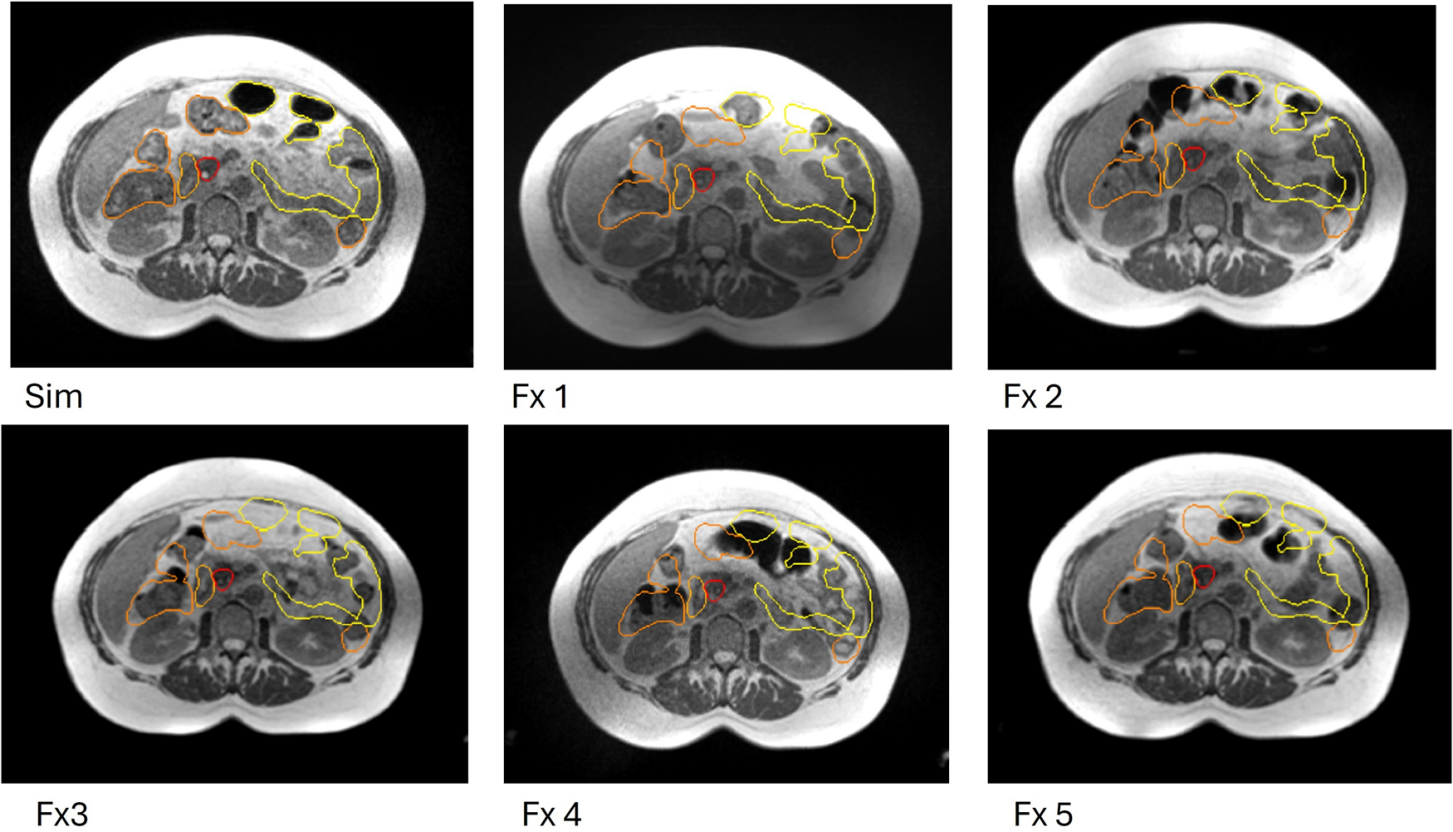
Axial MRI-Linac-weighted TRUFI images showing the contours of the target and organs at risk in a radiotherapy treatment plan, with simulation volumes (Sim) projected onto successive fractions (Fx1–Fx5). The images highlight the anatomical inter- and intra-variations affecting organ positioning during and between fractions. The figure shows the delineation of a pancreatic tumour (red line), loops of the small intestine (yellow line), and loops of the large intestine (orange line). This variability illustrates the potential of an online adaptive approach to reduce inaccuracies in treatment delivery based only on simulation imaging. MRI, magnetic resonance imaging; TRUFI, true fast imaging with steady-state free precession

It is well established that intra- and inter-fraction anatomical variations frequently occur throughout the course of RT, potentially compromising the precision of dose delivery and ultimately reducing tumour control efficacy [10]. Consequently, the conventional RT workflow warrants modernisation to enhance both treatment accuracy and efficiency. MRI-guided RT (MRIgRT) has emerged as a transformative modality by integrating real-time MRI with linear accelerators [46, 47]. This integration enables superior soft tissue contrast, intra-fraction visualisation, and daily online adaptive replanning. Particularly, adaptive MRIgRT allows for optimisation of the treatment plan based on real-time anatomical and functional changes while the patient remains in the treatment position, improving target coverage and sparing organs at risk, as shown in Fig. 2. In addition, continuous cine-MRI facilitates real-time motion tracking of mobile targets, such as the prostate and lung tumours, with automated beam gating when motion exceeds predefined thresholds—thus minimising geometric miss and enhancing treatment precision [46, 47]. Despite these advantages, widespread clinical implementation is challenged by extended treatment times, complex workflows, and the need for dedicated resources and training [48, 49].

## Step two: from imaging “for” RT to imaging “with” RT

### Real-time response monitoring

A further step toward cohesive care requires the incorporation of real-time imaging during RT delivery. It is now well known that, during treatment, the tumour response can consist of changes in size and/or biological characteristics, especially during long-course treatments [50]. Shifting the focus from post-treatment evaluation to intra-treatment adaptation allows clinicians to monitor tumour response dynamically, facilitating on-the-fly modifications to RT regimens [46]. Emerging MRIgRT systems exemplify this evolution, enabling both anatomical and functional imaging assessments within each treatment fraction.

It is well established—particularly in the immunotherapy context—that tumour size alone may not fully reflect the complexity of treatment response. For instance, the iRECIST criteria acknowledge that an apparent increase in tumour size may reflect inflammatory infiltration rather than true disease progression [51]. Similarly, the CHOI response criteria, developed for gastrointestinal stromal tumours, incorporate both tumour size and attenuation on CT imaging, using these parameters as surrogate biomarkers for therapeutic response [52]. Tumour heterogeneity further complicates response assessment, as it arises—at least in part—from the coexistence of multiple genetically distinct clonal populations within a single neoplasm [53].

In this scenario, functional and quantitative imaging techniques may offer the potential to detect early tumour microenvironment changes suggestive of treatment response or emerging radiation resistance—before alterations in tumour size become apparent. These imaging-derived features might support the identification of tumour subvolumes that could benefit from personalised dose modulation, including escalation or de-escalation strategies [54]. As an example, apparent diffusion coefficient (ADC) histogram parameters obtained from diffusion-weighted MRI have shown significant changes during concurrent chemo-RT in cervical cancer [55]. Preliminary results also suggest that mean ADC values of gross tumour volume may differ between responders and non-responders in patients with rectal cancer treated with MRIgRT. These findings indicate that ADC values can identify and quantify tissue changes in gross tumour volume during the RT treatment, supporting the development of dedicated decisional support systems [56].

### Identification and integration of imaging biomarkers

Imaging biomarkers are quantitative, objective features derived from medical images that reflect underlying biological and pathological processes or therapeutic responses [57]. They can successfully capture inter-patient variability in tumour biology and intra-patient changes over the course of treatment, supporting personalised care [58, 59]. Various imaging biomarkers have emerged from quantitative MRI, PET/CT, and CT techniques, with diffusion-weighted imaging, particularly ADC, being— as mentioned above—among the most established and promising for indirectly assessing tissue cellularity and showing early response in multiple cancer types [60–62]. Dynamic contrast-enhanced MRI biomarkers have also shown potential clinical application despite practical limitations associated with contrast administration and reproducibility [63].

The introduction of hybrid systems, which integrate an MRI with a linear accelerator (Linac), allows quantitative biomarkers to be obtained at each treatment fraction, thus capturing dynamic biological changes during RT. MRI-guided treatments enable quantitative imaging biomarkers to be acquired daily, which is practically not feasible on diagnostic MRI systems and provide valuable longitudinal information [64]. In this context, magnetic resonance fingerprinting (MRF) has emerged as a novel quantitative imaging technique, providing simultaneous T1 and T2 mapping with superior repeatability and reproducibility compared to conventional T1- and T2-weighted MRI [65]. Notably, MRF offers enhanced geometric accuracy, reduced susceptibility to imaging artefacts, and consistent statistical robustness, irrespective of scanner type or intensity normalisation algorithms.

When incorporated into MRI-linear accelerator workflows, MRF’s high inter-fractional temporal resolution enables the daily assessment of radiation-induced biological changes in both tumour and normal tissues, providing new insight into treatment dynamics [66]. Given these premises, standardisation and reproducibility of imaging biomarkers (*e.g*., acquisition protocols harmonisation across centres) remain crucial for their successful clinical integration. To address these challenges, dedicated efforts from collaborative groups such as the Quantitative Imaging Biomarkers Alliance (QIBA) have advanced standardisation processes, particularly focusing on ADC measurements [67, 68]. The next step is transitioning from technical to clinical validation. This could be achieved by incorporating validated biomarkers into prospective clinical trials, as exemplified by initiatives such as the THUNDER 2 study [69, 70].

Considerable research attention over the past decade has also been directed toward radiomics-derived biomarkers. However, despite the proliferation of research in this area, clinical applicability remains limited. This is partly attributable to insufficient alignment with specific clinical needs but is primarily due to persistent issues related to methodological heterogeneity and limited reproducibility [71–73]. One of the most promising applications of radiomics is the possibility to link imaging phenotypes to underlying genetic and molecular alterations—known as radiogenomics. Recent studies have demonstrated the capacity of radiomics to identify imaging phenotypes associated with distinct biological characteristics [74, 75]. Given that tumours are characterised by marked genetic heterogeneity, this heterogeneity may partially account for the observed variability in RT responses among patients with identical histological tumour types. For instance, the development of the genomic-adjusted radiation dose (GARD) model by Scott et al represents a significant advancement towards personalised medicine. By integrating a gene expression-based biomarker of tumour radiosensitivity (the radiosensitivity index (RSI)) with physical radiation dose, the genomic-adjusted radiation dose quantifies the biological effect of RT at an individual level, moving beyond the traditional “one-dose-fits-all” paradigm [76]. Identification of such information directly from imaging biomarkers could further expedite this individualisation process.

Looking forward, the integration of imaging biomarkers with data from other domains—including clinical parameters, genomics, and digital pathology—is essential to develop comprehensive, personalised predictive models. Several ongoing trials embody this multidisciplinary approach: the MOREOVER trial in rectal cancer (combining circulating tumour deoxyribonucleic acid, radiomics, and gut microbiota analyses), the MONDRIAN trial in lung cancer (integrating clinical, imaging, gene expression, and proteomic data), and the LANTERN trial in lung cancer (merging clinical, radiomics, and genomic data) [77–79]. Collectively, these efforts aim to create robust, individualised models to optimise patient outcomes.

## Step three: incorporation into clinical decision support systems

**Fig. 3:**
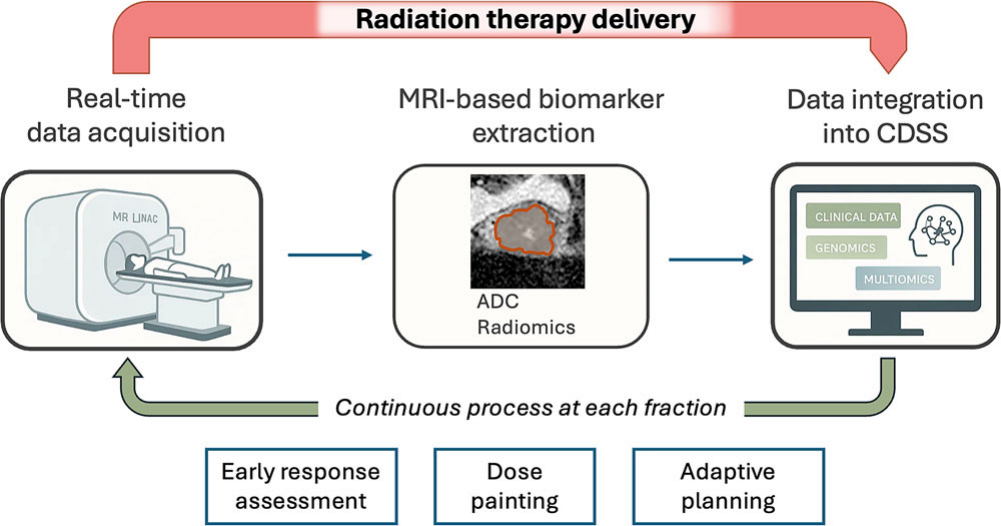
Continuous integration of real-time MRI data acquisition, possibly through advanced MRI-linear accelerator systems (MRI-Linac), could enable the extraction of MRI-based biomarkers (*e.g*., ADC, MRF, radiomics). These biomarkers might be combined with clinical, genomic, and multiomics data within AI-driven CDSS. Such an iterative approach at each treatment fraction could enhance personalised therapy by enabling earlier response assessments, precise dose painting, and adaptive planning strategies. ADC, apparent diffusion coefficient; AI, artificial intelligence; CDSS, clinical decision support systems; MRF, magnetic resonance fingerprint; MRI, magnetic resonance imaging

The progressive identification and validation of imaging-derived biomarkers naturally lead to their incorporation into clinical decision-making processes. Embedding these biomarkers within CDSS represents a crucial step toward translating complex, multidimensional data into actionable clinical strategies. Specifically, CDSS frameworks can facilitate real-time, data-driven treatment adaptations, including intra-treatment modifications, dose painting strategies, and toxicity risk predictions. A schematic representation of this upcoming future is illustrated in Fig. 3.

AI-powered CDSS platforms are particularly suitable for enhancing the clinical workflow. By automating the integration and interpretation of imaging biomarkers, alongside other patient-specific clinical and molecular data, AI-driven systems can accelerate decision-making and reduce interobserver variability. In doing so, they support the delivery of highly personalised and precise RT treatments, ensuring that each patient receives the most appropriate therapeutic approach based on their unique tumour and biological profile [80–82].

Moreover, the adoption of AI algorithms helps to overcome the limitations inherent to manual biomarker assessment, including the time-consuming nature of data extraction, the complexity of multi-parametric analysis, and potential interpretative biases. Recent research underscores the potential of such systems to streamline treatment planning and monitoring, making CDSS integration an indispensable component of future oncological care pathways [8, 72, 80, 83].

### Implementation roadmap for routine clinical use

Advanced imaging is increasingly integral to RT workflows; however, routine end-to-end clinical implementation has not yet been established. Achieving this requires inter-centre collaboration and secure data sharing to assemble harmonised, sufficiently large datasets for model development and validation. Accordingly, research priorities should shift from single-centre studies to large, multicentre prospective trials, and promote standardised acquisition/analysis protocols and quality assurance initiatives [71]. Health-economic evaluation is essential- —quantifying capital equipment, human resources, and development costs— as well as potential savings from reduced overtreatment and risk-adapted imaging and follow-up [84, 85]. Finally, because tailored (and potentially costly) strategies may not be universally applicable, implementation should prioritise indications and patients with the highest expected net clinical benefit [43].

## Conclusions

The integration of advanced imaging throughout the RT workflow is reshaping oncological care, shifting from standardised protocols to personalised strategies. Longitudinal quantitative imaging data acquisition during treatment potentially supports real-time tracking of tumour biology and on-table adaptation. In this context, imaging biomarkers can detect early microenvironmental changes and capture patient-specific temporal biological variability; when coupled with AI to automate labour-intensive tasks and integrate imaging, clinical and molecular data, these advances provide the basis for personalised RT. The clinical implementation of our vision, however, demands rigorous technical and clinical validation—together with health-economic evaluation—to ensure safe, reliable, and effective application in routine practice.

## Data Availability

Not applicable.

## Abbreviations

ADC: Apparent diffusion coefficient
AI: Artificial intelligence
CDSS: Clinical decision support systems
CT: Computed tomography
MDT: Multidisciplinary tumour board
MRF: Magnetic resonance fingerprinting
MRI: Magnetic resonance imaging
MRIgRT: MRI-guided radiotherapy
PET: Positron emission tomography
RT: Radiation therapy
sCT: Synthetic CT

## References

[1] Noble DJ, Ramaesh R, Brothwell M et al (2024) The evolving role of novel imaging techniques for radiotherapy planning. Clin Oncol 36:514–526. 10.1016/j.clon.2024.05.018 10.1016/j.clon.2024.05.018 38937188

[2] Thorwarth D (2015) Functional imaging for radiotherapy treatment planning: current status and future directions—a review. Br J Radiol 88:20150056. 10.1259/bjr.20150056 25827209 10.1259/bjr.20150056 PMC4628531

[3] Pillay B, Wootten AC, Crowe H et al (2016) The impact of multidisciplinary team meetings on patient assessment, management and outcomes in oncology settings: a systematic review of the literature. Cancer Treat Rev 42:56–72. 10.1016/j.ctrv.2015.11.007 26643552 10.1016/j.ctrv.2015.11.007

[4] Brannstrom F, Bjerregaard JK, Winbladh A et al (2015) Multidisciplinary team conferences promote treatment according to guidelines in rectal cancer. Acta Oncol 54:447–453. 10.3109/0284186X.2014.952387 25291075 10.3109/0284186X.2014.952387

[5] Hoffmann MS, Leslie LA, Jacobs RW et al (2016) Reducing the time from diagnosis to treatment of patients with stage II/III rectal cancer at a large public hospital. J Oncol Pract 12:e257–e262. 10.1200/JOP.2015.007484 26869658 10.1200/JOP.2015.007484 PMC4960466

[6] Hammer RD, Fowler D, Sheets LR, Siadimas A, Guo C, Prime MS (2020) Digital tumor board solutions have significant impact on case preparation. JCO Clin Cancer Inf 4:757–768. 10.1200/CCI.20.00029 10.1200/CCI.20.00029 PMC7469605 32816529

[7] Macchia G, Ferrandina G, Patarnello S et al (2021) Multidisciplinary tumor board smart virtual assistant in locally advanced cervical cancer: a proof of concept. Front Oncol 11:797454. 10.3389/fonc.2021.797454 35047408 10.3389/fonc.2021.797454 PMC8761664

[8] Fox T, Hughes F, Lai K et al (2022) Clinical decision support system for implementing care pathways in a global radiation oncology network. Int J Radiat Oncol Biol Phys 114:e111. 10.1016/j.ijrobp.2022.07.918

[9] Dona Lemus OM, Cao M, Cai B, Cummings M, Zheng D (2024) Adaptive radiotherapy: next-generation radiotherapy. Cancers (Basel) 16:1206. 10.3390/cancers16061206 10.3390/cancers16061206 PMC10968833 38539540

[10] Segedin B, Petric P (2016) Uncertainties in target volume delineation in radiotherapy—are they relevant and what can we do about them? Radiol Oncol 50:254–262. 10.1515/raon-2016-0023 27679540 10.1515/raon-2016-0023 PMC5024655

[11] Radici L, Ferrario S, Borca VC et al (2022) Implementation of a commercial deep learning-based auto segmentation software in radiotherapy: evaluation of effectiveness and impact on workflow. Life 12:2088. 10.3390/life12122088 36556455 10.3390/life12122088 PMC9782080

[12] Kleijnen JP, Van Asselen B, Burbach JP et al (2016) Evolution of motion uncertainty in rectal cancer: implications for adaptive radiotherapy. Phys Med Biol 61:1–11. 10.1088/0031-9155/61/1/1 26605518 10.1088/0031-9155/61/1/1

[13] Chang K, Beers AL, Bai HX et al (2019) Automatic assessment of glioma burden: a deep learning algorithm for fully automated volumetric and bidimensional measurement. Neuro Oncol 21:1412–1422. 10.1093/neuonc/noz106 31190077 10.1093/neuonc/noz106 PMC6827825

[14] Yu L, Zhao J, Xia F et al (2023) Technical note: first implementation of a one-stop solution of radiotherapy with full-workflow automation based on CT-linac combination. Med Phys 50:3117–3126. 10.1002/mp.16324 36842138 10.1002/mp.16324

[15] Ge Y, Wu QJ (2019) Knowledge-based planning for intensity-modulated radiation therapy: a review of data-driven approaches. Med Phys 46:2760–2775. 10.1002/mp.13526 30963580 10.1002/mp.13526 PMC6561807

[16] Zhou GQ, Yang YX, Yang X et al (2023) All-in-one online radiotherapy for nasopharyngeal carcinoma: preliminary results of treatment time, contouring accuracy, treatment plan quality and patient compliance. Int J Radiat Oncol Biol Phys 117:e636–e637. 10.1016/j.ijrobp.2023.06.2040

[17] Manganaro L, Lakhman Y, Bharwani N et al (2021) Staging, recurrence and follow-up of uterine cervical cancer using MRI: updated guidelines of the European Society of Urogenital Radiology after revised FIGO staging 2018. Eur Radiol 31:7802–7816. 10.1007/s00330-020-07632-9 33852049 10.1007/s00330-020-07632-9

[18] Beets-Tan RGH, Lambregts DMJ, Maas M et al (2018) Magnetic resonance imaging for clinical management of rectal cancer: updated recommendations from the 2016 European Society of Gastrointestinal and Abdominal Radiology (ESGAR) consensus meeting. Eur Radiol 28:1465–1475. 10.1007/s00330-017-5026-2 29043428 10.1007/s00330-017-5026-2 PMC5834554

[19] Weinreb JC, Barentsz JO, Choyke PL et al (2016) PI-RADS prostate imaging–reporting and data system: 2015, version 2. Eur Urol 69:16–40. 10.1016/j.eururo.2015.08.052 26427566 10.1016/j.eururo.2015.08.052 PMC6467207

[20] Niyazi M, Andratschke N, Bendszus M et al (2023) ESTRO-EANO guideline on target delineation and radiotherapy details for glioblastoma. Radiother Oncol 184:109663. 10.1016/j.radonc.2023.109663 37059335 10.1016/j.radonc.2023.109663

[21] Mayo ZS, Billena C, Suh JH, Lo SS, Chao ST (2024) The dilemma of radiation necrosis from diagnosis to treatment in the management of brain metastases. Neuro Oncol 26:S56–S65. 10.1093/neuonc/noad188 38437665 10.1093/neuonc/noad188 PMC10911797

[22] King AD, Mo FK, Yu KH et al (2010) Squamous cell carcinoma of the head and neck: diffusion-weighted MR imaging for prediction and monitoring of treatment response. Eur Radiol 20:2213–2220. 10.1007/s00330-010-1769-8 20309553 10.1007/s00330-010-1769-8

[23] Fu C, Bian D, Liu F, Feng X, Du W, Wang X (2012) The value of diffusion-weighted magnetic resonance imaging in assessing the response of locally advanced cervical cancer to neoadjuvant chemotherapy. Int J Gynecol Cancer 22:1037–1043. 10.1097/IGC.0b013e31825736d7 22683941 10.1097/IGC.0b013e31825736d7

[24] Lin G, Hsieh CY, Lai YC et al (2024) Hyperpolarized [1-(13)C]-pyruvate MRS evaluates immune potential and predicts response to radiotherapy in cervical cancer. Eur Radiol Exp 8:46. 10.1186/s41747-024-00445-1 38594558 10.1186/s41747-024-00445-1 PMC11003947

[25] Tang PLY, Romero AM, Nout RA et al (2024) Amide proton transfer-weighted CEST MRI for radiotherapy target delineation of glioblastoma: a prospective pilot study. Eur Radiol Exp 8:123. 10.1186/s41747-024-00523-4 39477835 10.1186/s41747-024-00523-4 PMC11525355

[26] Gunnlaugsson A, Persson E, Gustafsson C et al (2019) Target definition in radiotherapy of prostate cancer using magnetic resonance imaging only workflow. Phys Imaging Radiat Oncol 9:89–91. 10.1016/j.phro.2019.03.004 33458431 10.1016/j.phro.2019.03.004 PMC7807603

[27] De Pietro S, Di Martino G, Caroprese M et al (2024) The role of MRI in radiotherapy planning: a narrative review “from head to toe”. Insights Imaging 15:255. 10.1186/s13244-024-01799-1 39441404 10.1186/s13244-024-01799-1 PMC11499544

[28] Siemens Healthineers (2019) MR-only RT planning for the brain and pelvis with synthetic CT. Available via https://marketing.webassets.siemens-healthineers.com/4db6e75384fa9081/bec13c67d6a2/siemens-healthineers_syngo-via_white-paper-MR-based-Synthetic-CT.pdf. Accessed 28 Oct 2025

[29] Cusumano D, Dhont J, Boldrini L et al (2018) Predicting tumour motion during the whole radiotherapy treatment: a systematic approach for thoracic and abdominal lesions based on real time MR. Radiother Oncol 129:456–462. 10.1016/j.radonc.2018.07.025 30144955 10.1016/j.radonc.2018.07.025

[30] Boldrini L, Chiloiro G, Cusumano D et al (2023) Mesorectal motion evaluation in rectal cancer MR-guided radiotherapy: an exploratory study to quantify treatment margins. Radiat Oncol 18:4. 10.1186/s13014-022-02193-1 36604699 10.1186/s13014-022-02193-1 PMC9817323

[31] Cusumano D, Boldrini L, Dhont J et al (2021) Artificial intelligence in magnetic resonance guided radiotherapy: medical and physical considerations on state of art and future perspectives. Phys Med 85:175–191. 10.1016/j.ejmp.2021.05.010 34022660 10.1016/j.ejmp.2021.05.010

[32] Cusumano D, Placidi L, Teodoli S et al (2020) On the accuracy of bulk synthetic CT for MR-guided online adaptive radiotherapy. Radiol Med 125:157–164. 10.1007/s11547-019-01090-0 31591701 10.1007/s11547-019-01090-0

[33] Edmund JM, Nyholm T (2017) A review of substitute CT generation for MRI-only radiation therapy. Radiat Oncol 12:28. 10.1186/s13014-016-0747-y 28126030 10.1186/s13014-016-0747-y PMC5270229

[34] Persson E, Gustafsson C, Nordstrom F et al (2017) MR-OPERA: a multicenter/multivendor validation of magnetic resonance imaging-only prostate treatment planning using synthetic computed tomography images. Int J Radiat Oncol Biol Phys 99:692–700. 10.1016/j.ijrobp.2017.06.006 28843375 10.1016/j.ijrobp.2017.06.006

[35] Largent A, Barateau A, Nunes JC et al (2019) Pseudo-CT generation for MRI-only radiation therapy treatment planning: comparison among patch-based, atlas-based, and bulk density methods. Int J Radiat Oncol Biol Phys 103:479–490. 10.1016/j.ijrobp.2018.10.002 30336265 10.1016/j.ijrobp.2018.10.002

[36] Largent A, Barateau A, Nunes JC et al (2019) Comparison of deep learning-based and patch-based methods for pseudo-CT generation in MRI-based prostate dose planning. Int J Radiat Oncol Biol Phys 105:1137–1150. 10.1016/j.ijrobp.2019.08.049 31505245 10.1016/j.ijrobp.2019.08.049

[37] Cusumano D, Lenkowicz J, Votta C et al (2020) A deep learning approach to generate synthetic CT in low field MR-guided adaptive radiotherapy for abdominal and pelvic cases. Radiother Oncol 153:205–212. 10.1016/j.radonc.2020.10.018 33075394 10.1016/j.radonc.2020.10.018

[38] Autret D, Guillerminet C, Roussel A, Cossec-Kerloc’h E, Dufreneix S (2023) Comparison of four synthetic CT generators for brain and prostate MR-only workflow in radiotherapy. Radiat Oncol 18:146. 10.1186/s13014-023-02336-y 37670397 10.1186/s13014-023-02336-y PMC10478301

[39] Huijben EMC, Terpstra ML, Galapon AJ et al (2024) Generating synthetic computed tomography for radiotherapy: Synthrad2023 challenge report. Med Image Anal 97:103276. 10.1016/j.media.2024.103276 39068830 10.1016/j.media.2024.103276

[40] Persson E, Jamtheim Gustafsson C, Ambolt P et al (2020) MR-protect: clinical feasibility of a prostate MRI-only radiotherapy treatment workflow and investigation of acceptance criteria. Radiat Oncol 15:77. 10.1186/s13014-020-01513-7 32272943 10.1186/s13014-020-01513-7 PMC7147064

[41] Ahangari S, Hansen NL, Olin AB et al (2021) Toward PET/MRI as one-stop shop for radiotherapy planning in cervical cancer patients. Acta Oncol 60:1045–1053. 10.1080/0284186X.2021.193616442 34107847 10.1080/0284186X.2021.1936164

[42] Villegas F, Dal Bello R, Alvarez-Andres E et al (2024) Challenges and opportunities in the development and clinical implementation of artificial intelligence based synthetic computed tomography for magnetic resonance only radiotherapy. Radiother Oncol 198:110387. 10.1016/j.radonc.2024.110387 38885905 10.1016/j.radonc.2024.110387

[43] Goudschaal K, Beeksma F, Boon M et al (2021) Accuracy of an MR-only workflow for prostate radiotherapy using semi-automatically burned-in fiducial markers. Radiat Oncol 16:37. 10.1186/s13014-021-01768-8 33608008 10.1186/s13014-021-01768-8 PMC7893889

[44] Tanadini-Lang S, Budgell G, Bohoudi O et al (2023) An ESTRO-ACROP guideline on quality assurance and medical physics commissioning of online MRI guided radiotherapy systems based on a consensus expert opinion. Radiother Oncol 181:109504. 10.1016/j.radonc.2023.109504 36736592 10.1016/j.radonc.2023.109504

[45] Hall WA, Paulson ES, van der Heide UA et al (2019) The transformation of radiation oncology using real-time magnetic resonance guidance: a review. Eur J Cancer 122:42–52. 10.1016/j.ejca.2019.07.021 31614288 10.1016/j.ejca.2019.07.021 PMC8447225

[46] Benitez CM, Chuong MD, Kunzel LA, Thorwarth D (2024) MRI-guided adaptive radiation therapy. Semin Radiat Oncol 34:84–91. 10.1016/j.semradonc.2023.10.013 38105097 10.1016/j.semradonc.2023.10.013

[47] Nishioka S, Okamoto H, Chiba T et al (2022) Identifying risk characteristics using failure mode and effect analysis for risk management in online magnetic resonance-guided adaptive radiation therapy. Phys Imaging Radiat Oncol 23:1–7. 10.1016/j.phro.2022.06.002 35712526 10.1016/j.phro.2022.06.002 PMC9194450

[48] Rippke C, Schrenk O, Renkamp CK et al (2022) Quality assurance for on-table adaptive magnetic resonance guided radiation therapy: a software tool to complement secondary dose calculation and failure modes discovered in clinical routine. J Appl Clin Med Phys 23:e13523. 10.1002/acm2.13523 35019212 10.1002/acm2.13523 PMC8906229

[49] Carlos-Reyes A, Muniz-Lino MA, Romero-Garcia S, Lopez-Camarillo C, Hernandez-de la Cruz ON (2021) Biological adaptations of tumor cells to radiation therapy. Front Oncol 11:718636. 10.3389/fonc.2021.718636 34900673 10.3389/fonc.2021.718636 PMC8652287

[50] Seymour L, Bogaerts J, Perrone A et al (2017) iRECIST: guidelines for response criteria for use in trials testing immunotherapeutics. Lancet Oncol 18:e143–e152. 10.1016/S1470-2045(17)30074-8 28271869 10.1016/S1470-2045(17)30074-8 PMC5648544

[51] Choi H, Charnsangavej C, Faria SC et al (2007) Correlation of computed tomography and positron emission tomography in patients with metastatic gastrointestinal stromal tumor treated at a single institution with imatinib mesylate: proposal of new computed tomography response criteria. J Clin Oncol 25:1753–1759. 10.1200/JCO.2006.07.3049 17470865 10.1200/JCO.2006.07.3049

[52] Cassidy JW, Bruna A (2017) Tumor heterogeneity. In: Uthamanthil R, Tinkey P (eds) Patient derived tumor xenograft models, 1st edn. Academic Press, Cambridge

[53] Garcia-Figueiras R, Baleato-Gonzalez S, Luna A et al (2024) How imaging advances are defining the future of precision radiation therapy. Radiographics 44:e230152. 10.1148/rg.230152 38206833 10.1148/rg.230152

[54] Meng J, Zhu L, Zhu L et al (2016) Apparent diffusion coefficient histogram shape analysis for monitoring early response in patients with advanced cervical cancers undergoing concurrent chemo-radiotherapy. Radiat Oncol 11:141. 10.1186/s13014-016-0715-6 27770816 10.1186/s13014-016-0715-6 PMC5075415

[55] Nardini M, Mazzoni LN, Galetto M et al (2023) MO-04.7—online adaptive MRGRT diffusion weighted driven: a phantom study for the clinical implementation. Phys Med 115:102814. 10.1016/j.ejmp.2023.102814

[56] Kessler LG, Barnhart HX, Buckler AJ et al (2015) The emerging science of quantitative imaging biomarkers terminology and definitions for scientific studies and regulatory submissions. Stat Methods Med Res 24:9–26. 10.1177/0962280214537333 24919826 10.1177/0962280214537333

[57] Baumann M, Krause M, Overgaard J et al (2016) Radiation oncology in the era of precision medicine. Nat Rev Cancer 16:234–249. 10.1038/nrc.2016.18 27009394 10.1038/nrc.2016.18

[58] Garcia-Figueiras R, Baleato-Gonzalez S, Padhani AR et al (2019) How clinical imaging can assess cancer biology. Insights Imaging 10:28. 10.1186/s13244-019-0703-0 30830470 10.1186/s13244-019-0703-0 PMC6399375

[59] Trada Y, Keall P, Jameson M et al (2023) Changes in serial multiparametric MRI and FDG-PET/CT functional imaging during radiation therapy can predict treatment response in patients with head and neck cancer. Eur Radiol 33:8788–8799. 10.1007/s00330-023-09843-2 37405500 10.1007/s00330-023-09843-2 PMC10667402

[60] Winter RM, Boeke S, Leibfarth S et al (2025) Clinical validation of a prognostic preclinical magnetic resonance imaging biomarker for radiotherapy outcome in head-and-neck cancer. Radiother Oncol 204:110702. 10.1016/j.radonc.2024.110702 39733969 10.1016/j.radonc.2024.110702

[61] Liang C, Wang W, Yang G et al (2024) Utility of interim apparent diffusion coefficient value in predicting treatment response among patients with locally advanced cervical cancer treated with radiotherapy. Clin Transl Radiat Oncol 48:100827. 10.1016/j.ctro.2024.100827 39192879 10.1016/j.ctro.2024.100827 PMC11347826

[62] Otazo R, Lambin P, Pignol JP et al (2021) MRI-guided radiation therapy: an emerging paradigm in adaptive radiation oncology. Radiology 298:248–260. 10.1148/radiol.2020202747 33350894 10.1148/radiol.2020202747 PMC7924409

[63] Kooreman ES, van Houdt PJ, Keesman R et al (2020) ADC measurements on the Unity MR-linac—a recommendation on behalf of the Elekta Unity MR-linac consortium. Radiother Oncol 153:106–113. 10.1016/j.radonc.2020.09.046 33017604 10.1016/j.radonc.2020.09.046 PMC8327388

[64] Coppo S, Mehta BB, McGivney D et al (2016) Overview of magnetic resonance fingerprinting. MAGNETOM Flash 1:12–21. Available via https://cdn0.scrvt.com/39b415fb07de4d9656c7b516d8e2d907/1800000003051768/c5008329ed3d/Overview-of-Magnetic-Resonance-Fingerprinting_1800000003051768.pdf. Accessed 28 Oct 2025

[65] Chen Y, Lu L, Zhu T, Ma D (2022) Technical overview of magnetic resonance fingerprinting and its applications in radiation therapy. Med Phys 49:2846–2860. 10.1002/mp.15254 34633687 10.1002/mp.15254 PMC12911327

[66] Van Houdt PJ, Saeed H, Thorwarth D et al (2021) Integration of quantitative imaging biomarkers in clinical trials for MR-guided radiotherapy: conceptual guidance for multicentre studies from the MR-linac consortium imaging biomarker working group. Eur J Cancer 153:64–71. 10.1016/j.ejca.2021.04.041 34144436 10.1016/j.ejca.2021.04.041 PMC8340311

[67] Boss MA, Malyarenko D, Partridge S et al (2024) The QIBA profile for diffusion-weighted MRI: apparent diffusion coefficient as a quantitative imaging biomarker. Radiology 313:e233055. 10.1148/radiol.233055 39377680 10.1148/radiol.233055 PMC11537247

[68] Chiloiro G, Cusumano D, Boldrini L et al (2022) Thunder 2: theragnostic utilities for neoplastic diseases of the rectum by MRI guided radiotherapy. BMC Cancer 22:67. 10.1186/s12885-021-09158-9 35033008 10.1186/s12885-021-09158-9 PMC8760695

[69] Van Houdt PJ, Li S, Yang Y, Van Der Heide UA (2024) Quantitative MRI on MR-linacs: towards biological image-guided adaptive radiotherapy. Semin Radiat Oncol 34:107–119. 10.1016/j.semradonc.2023.10.010 38105085 10.1016/j.semradonc.2023.10.010

[70] Whybra P, Zwanenburg A, Andrearczyk V et al (2024) The image biomarker standardization initiative: standardized convolutional filters for reproducible radiomics and enhanced clinical insights. Radiology 310:e231319. 10.1148/radiol.231319 38319168 10.1148/radiol.231319 PMC10902595

[71] Russo L, Bottazzi S, Sala E (2024) Artificial intelligence in female pelvic oncology: tailoring applications to clinical needs. Eur Radiol 34:4038–4040. 10.1007/s00330-023-10455-z 37989917 10.1007/s00330-023-10455-z

[72] Russo L, Charles-Davies D, Bottazzi S, Sala E, Boldrini L (2024) Radiomics for clinical decision support in radiation oncology. Clin Oncol 36:e269–e281. 10.1016/j.clon.2024.03.003 10.1016/j.clon.2024.03.003 38548581

[73] Su GH, Xiao Y, You C et al (2023) Radiogenomic-based multiomic analysis reveals imaging intratumor heterogeneity phenotypes and therapeutic targets. Sci Adv 9:eadf0837. 10.1126/sciadv.adf0837 37801493 10.1126/sciadv.adf0837 PMC10558123

[74] Crispin-Ortuzar M, Woitek R, Reinius MAV et al (2023) Integrated radiogenomics models predict response to neoadjuvant chemotherapy in high grade serous ovarian cancer. Nat Commun 14:6756. 10.1038/s41467-023-41820-7 37875466 10.1038/s41467-023-41820-7 PMC10598212

[75] Scott JG, Sedor G, Ellsworth P et al (2021) Pan-cancer prediction of radiotherapy benefit using genomic-adjusted radiation dose (GARD): a cohort-based pooled analysis. Lancet Oncol 22:1221–1229. 10.1016/S1470-2045(21)00347-8 34363761 10.1016/S1470-2045(21)00347-8 PMC12818176

[76] Boldrini L, Chiloiro G, Di Franco S et al (2024) MOREOVER: multiomics MR-guided radiotherapy optimization in locally advanced rectal cancer. Radiat Oncol 19:94. 10.1186/s13014-024-02492-9 39054479 10.1186/s13014-024-02492-9 PMC11271028

[77] Volpe S, Zaffaroni M, Piperno G et al (2023) Multi-omics integrative modelling for stereotactic body radiotherapy in early-stage non-small cell lung cancer: clinical trial protocol of the MONDRIAN study. BMC Cancer 23:1236. 10.1186/s12885-023-11701-9 38102575 10.1186/s12885-023-11701-9 PMC10722797

[78] Lococo F, Boldrini L, Diepriye CD et al (2023) Lung cancer multi-omics digital human avatars for integrating precision medicine into clinical practice: the LANTERN study. BMC Cancer 23:540. 10.1186/s12885-023-10997-x 37312079 10.1186/s12885-023-10997-x PMC10262371

[79] Valdes G, Simone CB 2nd, Chen J et al (2017) Clinical decision support of radiotherapy treatment planning: a data-driven machine learning strategy for patient-specific dosimetric decision making. Radiother Oncol 125:392–397. 10.1016/j.radonc.2017.10.014 29162279 10.1016/j.radonc.2017.10.014

[80] Yu SH, Kim MS, Chung HS et al (2021) Early experience with Watson for oncology: a clinical decision-support system for prostate cancer treatment recommendations. World J Urol 39:407–413. 10.1007/s00345-020-03214-y 32335733 10.1007/s00345-020-03214-y

[81] Nafees A, Khan M, Chow R et al (2023) Evaluation of clinical decision support systems in oncology: an updated systematic review. Crit Rev Oncol Hematol 192:104143. 10.1016/j.critrevonc.2023.104143 37742884 10.1016/j.critrevonc.2023.104143

[82] Cesario A, Simone I, Paris I et al (2021) Development of a digital research assistant for the management of patients’ enrollment in oncology clinical trials within a research hospital. J Pers Med 11:244. 10.3390/jpm11040244 33801668 10.3390/jpm11040244 PMC8066078

[83] Khanna NN, Maindarkar MA, Viswanathan V et al (2022) Economics of artificial intelligence in healthcare: diagnosis vs. treatment. Healthcare 10:2493. 10.3390/healthcare10122493 36554017 10.3390/healthcare10122493 PMC9777836

[84] Magnani CJ, Bievre N, Baker LC, Brooks JD, Blayney DW, Hernandez-Boussard T (2021) Real-world evidence to estimate prostate cancer costs for first-line treatment or active surveillance. Eur Urol Open Sci 23:20–29. 10.1016/j.euros.2020.11.004 33367287 10.1016/j.euros.2020.11.004 PMC7751921

[85] Powell A, Batumalai V, Wong K et al (2024) Cost-outcome of radiotherapy for local control and overall survival benefits in breast cancer. Clin Oncol 36:651–657. 10.1016/j.clon.2024.07.007 10.1016/j.clon.2024.07.007 39117508

